# Diagnostic Accuracy of Cryptococcal Antigen Lateral Flow Assay Compared With Microscopy and Culture in Suspected Cryptococcal Meningitis

**DOI:** 10.7759/cureus.113722

**Published:** 2026-07-31

**Authors:** Kumar Arpit, Anju Mahor, Suneel Ahirwar, Manish Purohit

**Affiliations:** 1 Microbiology, Mahatma Gandhi Memorial Medical College, Indore, IND

**Keywords:** cryptococcal meningitis, culture, diagnostic accuracy, lateral flow assay, point-of-care test (poct)

## Abstract

Introduction

Cryptococcosis is a serious fungal infection that can occur in both immunocompromised and immunocompetent individuals and may be fatal when diagnosis is delayed. Rapid and reliable point-of-care testing can facilitate early diagnosis and timely initiation of treatment. Therefore, this study aimed to assess the diagnostic performance of the lateral flow assay (LFA) for cryptococcal meningitis, using microscopy and fungal culture as reference methods.

Materials and methods

This comparative observational study was conducted at Mahatma Gandhi Memorial Medical College in Indore, India, over a period of six months (April-September 2025). The cerebrospinal fluid (CSF) samples (n=60) from patients with suspected meningitis were considered for the study. All CSF samples were examined using India ink preparation, and culture was done on Sabouraud dextrose agar, whereas LFA was done on both CSF and serum samples using Biosynex CryptoPS (Biosynex SA, Illkirch-Graffenstaden, France). The results of microscopy and culture were then compared with LFA.

Results

Among 60 suspected meningitis patients, eight grew *Cryptococcus* in culture which was regarded as the gold standard for confirming the diagnosis of cryptococcal meningitis. Direct microscopy had a specificity of 100%. However, its sensitivity was less, i.e., 62.5%. LFA was also performed on both CSF and serum of the same patients, further yielding good sensitivity, specificity, negative predictive value (NPV), and positive predictive value (PPV), thus confirming its reliability as a rapid diagnostic tool.

Conclusion

LFA emerges as a game-changer in the rapid diagnosis of cryptococcal meningitis. It offers excellent concordance with culture while offering superior speed and bedside utility over traditional methods. As a true point-of-care innovation, LFA bridges the gap between clinical suspicion and early treatment initiation, a step that can be life-saving in resource-limited settings.

## Introduction

Cryptococcal meningitis is a life-threatening invasive fungal infection caused predominantly by *Cryptococcus neoformans* and *Cryptococcus gattii*. It remains a major cause of morbidity and mortality among both immunocompromised and, increasingly, immunocompetent individuals, particularly when diagnosis and treatment are delayed. Despite advances in antifungal therapy, cryptococcal meningitis continues to account for a substantial proportion of fungal meningitis cases worldwide, with a high case-fatality rate, especially in low- and middle-income countries where access to rapid diagnostics is limited [1-3].

The definitive diagnosis of cryptococcal meningitis traditionally relies on cerebrospinal fluid (CSF) fungal culture, which is considered the gold standard because of its high specificity. However, culture requires several days to yield results and may show reduced sensitivity in patients with a low fungal burden or those who have received prior antifungal therapy. Direct microscopic examination using India ink preparation is inexpensive and rapid but has limited sensitivity, particularly in early disease and in patients with low concentrations of cryptococcal organisms [4-6].

The detection of cryptococcal antigen (CrAg) by lateral flow assay (LFA) has emerged as a significant advancement in the diagnosis of cryptococcosis. LFA is a rapid, simple, and highly sensitive immunochromatographic assay that can detect CrAg in CSF and serum within minutes without requiring sophisticated laboratory infrastructure. Owing to its excellent diagnostic performance, ease of use, and suitability for point-of-care testing, the World Health Organization (WHO) recommends CrAg LFA for the diagnosis and screening of cryptococcal disease, particularly in resource-limited settings [7-9].

Considering the need for rapid and accurate diagnosis of cryptococcal meningitis, this study was undertaken to evaluate the diagnostic accuracy of LFA in comparison with conventional diagnostic methods, namely, CSF culture (gold standard) and direct microscopy (India ink preparation), among patients with suspected meningitis. The findings of this study may help guide the selection of effective diagnostic strategies for the early detection and prompt management of cryptococcal meningitis, thereby reducing disease-associated morbidity and mortality.

## Materials and methods

Study design and setting

A prospective, comparative diagnostic accuracy study was conducted in the Department of Microbiology of Mahatma Gandhi Memorial Medical College in Indore, India, over six months, from April to September 2025. The study evaluated the diagnostic performance of the cryptococcal antigen LFA, direct microscopy, and fungal culture for detecting cryptococcal meningitis in CSF samples.

Study population

The study included CSF specimens received in the microbiology laboratory from patients with clinical and/or biochemical suspicion of meningitis. Clinical suspicion was based on features such as headache, fever, neck stiffness, altered sensorium, seizures, or signs of raised intracranial pressure. Biochemical suspicion included abnormalities such as increased CSF protein, decreased CSF glucose, or CSF pleocytosis. A total of 60 eligible CSF specimens were examined during the study period.

Sampling technique

A consecutive sampling technique was used. All CSF specimens received during the study period that fulfilled the predefined eligibility criteria were included until the required sample size was achieved. This approach was used to reduce selection bias and ensure that the study population represented patients routinely investigated for suspected meningitis at the institution. Only one CSF specimen per patient was included in the primary analysis. Repeat samples from the same episode of illness were excluded unless repeat-sample analysis formed part of the predefined study protocol.

Inclusion criteria

CSF specimens obtained from patients of all age groups and both sexes with clinical and/or biochemical suspicion of meningitis were included, provided they were received in adequate quantity for microscopy, fungal culture, and cryptococcal antigen LFA, were collected and transported according to standard laboratory protocols, and had the required clinical and demographic information available.

Exclusion criteria

Grossly contaminated, improperly labelled, or lipid-contaminated CSF specimens, specimens with characteristics likely to interfere with test interpretation, specimens received in leaking or inappropriate containers, and specimens of insufficient volume to perform all three diagnostic tests were excluded.

Sample size determination

All consecutive eligible CSF specimens received during the six-month study period were included. A total of 60 specimens fulfilled the eligibility criteria and were analyzed.

Specimen collection and processing

CSF was collected aseptically by the treating clinical team through lumbar puncture as part of routine patient management. No additional lumbar puncture was performed solely for this study. Specimens were transported promptly to the microbiology laboratory in sterile, leak-proof containers and processed according to the standard operating procedure.

Where adequate volume was available, the specimen was divided into portions for direct microscopy, fungal culture, and cryptococcal antigen detection. When centrifugation was performed, the sediment was used for microscopy and culture, while the appropriate specimen fraction was used for antigen testing according to the manufacturer's instructions.

Direct microscopy

Direct microscopic examination of each CSF specimen was performed using India ink preparation. For India ink examination, a drop of CSF sediment was mixed with India ink on a clean glass slide, covered with a coverslip, and examined under the microscope. The demonstration of round or oval budding yeast cells surrounded by a clear halo against a dark background was considered positive for encapsulated yeast consistent with *Cryptococcus*.

Fungal culture

Each CSF specimen was inoculated onto Sabouraud dextrose agar and incubated at 37°C. The culture plates were examined daily for up to 14 days for evidence of fungal growth. Colonies suspected to be *Cryptococcus *were identified on the basis of colony morphology, demonstration of the capsule by India ink preparation, urease activity, phenoloxidase-mediated melanin production, carbohydrate assimilation patterns, and other standard laboratory identification methods available during the study. A culture was considered positive when *Cryptococcus *species was isolated and confirmed by the predefined identification protocol.

Cryptococcal antigen LFA

Cryptococcal antigen detection was performed using LFA according to the kit literature. The required quantity of CSF was applied to the test device or sample-processing system supplied with the kit Biosynex CryptoPS (Biosynex SA, Illkirch-Graffenstaden, France). The test was interpreted within the manufacturer-recommended time interval. The appearance of a valid control line indicated that the test had functioned correctly. The appearance of the relevant test line, along with the control line, was interpreted as a positive result. Tests without a control line were considered invalid and were repeated using a new device whenever sufficient specimen was available.

Clinical and epidemiological data

Relevant demographic and clinical information was obtained from laboratory request forms and available clinical records. Variables assessed included age, sex, immune status, HIV infection, corticosteroid or immunosuppressive therapy, malignancy, organ transplantation, diabetes mellitus, tuberculosis, prior antifungal exposure, and other recognized clinical risk factors. Patient-identifying details were removed before statistical analysis, and each specimen was assigned a unique study code.

Outcome measures

The primary outcome was the diagnostic performance of the cryptococcal antigen LFA for detecting cryptococcal meningitis, using CSF fungal culture as the reference standard. Secondary outcomes included the diagnostic performance of India ink examination, agreement between LFA, microscopy, and culture, and time required to obtain the test results.

Statistical analysis

Data were entered into Microsoft Excel (Microsoft Corporation, Redmond, Washington, United States) and analyzed using IBM SPSS Statistics for Windows, Version 25.0 (IBM Corp., Armonk, New York, United States).

Two-by-two contingency tables were prepared for microscopy and LFA against CSF culture. The following diagnostic parameters were calculated: $\mathrm{Specificity}=\frac{\text{True Negatives}}{\text{True Negatives}+\text{False Positives}}\times100$. Diagnostic accuracy was calculated as follows: $\mathrm{Accuracy}=\frac{\text{True Positives}+\text{True Negatives}}{\text{Total Number of Specimens}}\times100$.

Results were reported as percentages with 95% CI where applicable. Agreement between diagnostic methods was assessed using Cohen's kappa coefficient, when performed. Categorical variables were presented as frequencies and percentages, while continuous variables were expressed as mean with standard deviation or median with interquartile range, depending on their distribution. A two-sided p-value of less than 0.05 was considered statistically significant.

Ethical considerations

Ethical approval was obtained from the Ethics and Scientific Review Committee of Mahatma Gandhi Memorial Medical College and Maharaja Yeshwantrao Hospital (approval number: EC/MGM/MAY-24/06; date: May 13, 2024). CSF specimens were collected as part of routine clinical evaluation, and no additional lumbar puncture was performed exclusively for the study. Confidentiality was maintained by anonymizing patient information before analysis. Written informed consent was obtained from the participants or their legally authorized representatives, where required under the approved protocol.

## Results

Characteristics of the study samples

A total of 60 CSF specimens from 60 patients with clinically and/or biochemically suspected meningitis were analyzed. All specimens were of adequate volume and quality for microscopy, fungal culture, and CrAg LFA. Culture confirmed cryptococcal meningitis in eight of 60 patients (13.3%). Direct microscopy detected *Cryptococcus *in five of 60 patients (8.3%), corresponding to five of the eight culture-positive cases and a sensitivity of 62.5%. All five microscopy-positive specimens were culture positive, with no false-positive microscopy results. The CrAg LFA detected all eight culture-confirmed cases (13.3%) and produced no false-positive or false-negative results, resulting in 100% sensitivity and 100% specificity in the present study population.

With respect to age distribution, the largest proportion of patients belonged to the 30-50-year age group, accounting for 30 patients (50%). Patients older than 50 years constituted 18 cases (30%), while those younger than 30 years accounted for 12 cases (20%). This indicates that most suspected cases were concentrated in the middle-aged population. The study population showed a male predominance, with 36 males (60%) and 24 females (40%). Among the major immune-related and clinical risk factors, diabetes mellitus was the most common, observed in 13 patients (21.7%). HIV infection was present in eight patients (13.3%), while immunosuppressive therapy was documented in six patients (10%). Decompensated liver disease was noted in five patients (8.3%), prior antifungal exposure in four patients (6.7%), malignancy in two patients (3.3%), and organ transplantation in one patient (1.7%). Other recognized clinical risk factors were less frequent. Coronary artery disease was present in four patients (6.7%), whereas cerebrovascular accident and tuberculosis were each identified in three patients (5%). Since some patients had more than one underlying condition, the total frequency of risk factors exceeds the total number of culture-confirmed cases (Table 1).

**Table 1:** Demographic, clinical risk factor, and diagnostic profile of the study population (n=60) CSF: cerebrospinal fluid

Variable	n	%
Study population		
Total CSF specimens/patients analyzed	60	100
Culture-confirmed cryptococcal meningitis	8	13.3
Age distribution		
<30 years	12	20
30-50 years	30	50
>50 years	18	30
Sex		
Male	36	60
Female	24	40
Immune status/major risk factors		
Diabetes mellitus	13	21.7
HIV infection	8	13.3
Immunosuppressive therapy	6	10
Decompensated liver disease	5	8.3
Malignancy	2	3.3
Organ transplantation	1	1.7
Prior antifungal exposure	4	6.7
Other recognized clinical risk factors		
Coronary artery disease	4	6.7
Cerebrovascular accident	3	5
Tuberculosis	3	5

**Figure 1:**
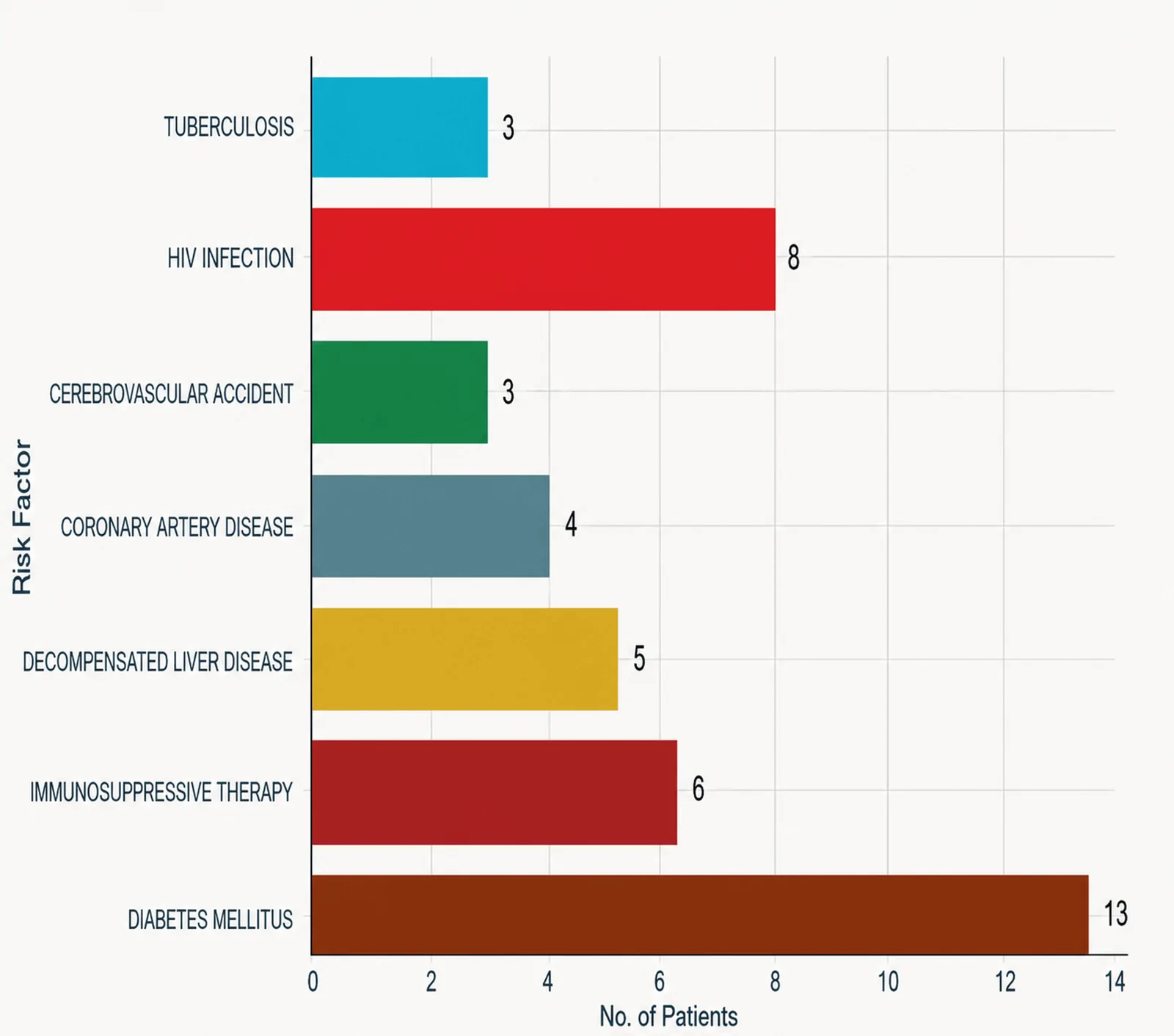
Distribution of underlying risk factors/comorbid conditions among suspected meningitis patients (n=60)

The pattern of risk factors demonstrated that diabetes mellitus was the most frequently encountered condition among the study participants, indicating its prominent association with the patient profile. HIV infection and immunosuppressive therapy also contributed substantially, suggesting that impaired host immunity was an important feature in this cohort. Decompensated liver disease and coronary artery disease were identified in a moderate number of patients, while tuberculosis and cerebrovascular accident were comparatively less common (Figure 1).

Fungal culture was considered the reference standard for evaluating the diagnostic performance of direct microscopy and the CrAg LFA. As the reference method, culture showed 100% sensitivity, specificity, positive predictive value (PPV), and negative predictive value (NPV) by definition. Direct microscopy demonstrated a sensitivity of 62.5%, indicating that it detected only five of the eight culture-confirmed cases and missed three positive cases. However, its specificity and PPV were both 100%, showing that all microscopy-positive results were true positives and that no false-positive result occurred. The NPV was 94.5%, suggesting that most microscopy-negative specimens were truly negative, although a negative microscopy result could not reliably exclude cryptococcal meningitis.

In comparison, the CrAg LFA demonstrated 100% sensitivity, specificity, PPV, and NPV. It correctly identified all culture-positive as well as all culture-negative specimens, with no false-positive or false-negative results. These findings indicate that the LFA performed better than direct microscopy in the present study and provided diagnostic accuracy comparable to culture while offering a much shorter turnaround time. The detailed diagnostic performance of the three methods is presented in Table 2.

**Table 2:** Sensitivity, specificity, and predictive values for culture and direct microscopy in CSF samples (n=60) PPV: positive predictive value; NPV: negative predictive value; CSF: cerebrospinal fluid

Tests	Sensitivity (%)	Specificity (%)	PPV (%)	NPV (%)
Culture (gold standard)	100	100	100	100
Direct microscopy	62.5	100	100	94.5
Lateral flow assay	100	100	100	100

Overall, the CrAg LFA showed excellent diagnostic performance, correctly identifying all culture-confirmed cases without producing any false-positive or false-negative results. In contrast, direct microscopy missed three culture-positive cases because of its lower sensitivity. These findings indicate that CrAg LFA is a rapid, reliable, and highly accurate diagnostic tool that may support the early recognition of cryptococcal meningitis and the timely initiation of appropriate antifungal treatment.

## Discussion

The present study evaluated the diagnostic performance of the CrAg LFA in comparison with conventional diagnostic methods for cryptococcal meningitis. Culture remained the reference standard and identified eight confirmed cases of cryptococcal meningitis. Fungal culture was used as the reference standard because isolation of *Cryptococcus *from CSF provides definitive microbiological evidence of infection. However, culture has important practical and diagnostic limitations. The organism may require several days to produce visible growth, thereby delaying the confirmation and initiation of targeted treatment. Culture sensitivity is also affected by the volume of CSF inoculated, fungal burden, previous antifungal therapy, and specimen-processing methods. In a large multisite validation study, Boulware et al. reported that culture sensitivity increased from 82.4% when 10 µL of CSF was inoculated to 94.2% when 100 µL was used [8]. Thus, culture-negative cryptococcal meningitis may occur, particularly in patients with a low organism burden or prior antifungal exposure. Although fungal culture demonstrated excellent diagnostic accuracy, its major limitation is the prolonged turnaround time and the requirement for specialized laboratory infrastructure, making it less suitable for rapid clinical decision-making. Similar observations have been reported previously [3,5].

Direct microscopy using India ink preparation demonstrated a sensitivity of 62.5% and a specificity of 100% in the present study. The reduced sensitivity is comparable to previous studies, which have shown that India ink examination has limited sensitivity in patients with low fungal burden or those receiving antifungal therapy [4,9].

These findings are consistent with studies by Jarvis et al., Hansen et al., and Boulware et al., which reported sensitivities and specificities exceeding 99% for CrAg LFA [6-8]. The assay provides rapid results within minutes, requires minimal laboratory infrastructure, and can be performed at the point of care, making it particularly valuable in resource-limited settings where delayed diagnosis contributes significantly to mortality [2,10].

The high NPV observed in this study indicates that a negative LFA result can reliably exclude cryptococcal meningitis in patients with suspected disease. This supports WHO recommendations advocating CrAg LFA as a first-line diagnostic and screening tool in high-risk patients [2].

The present study also identified diabetes mellitus and immunosuppression as the most common underlying risk factors among patients with cryptococcal meningitis. While cryptococcosis has classically been associated with HIV infection and other immunocompromised states, recent evidence suggests that diabetes mellitus is an increasingly recognized risk factor because of impaired host immune responses [1,11,12].

Chisale et al., in a comparative study of 265 specimens, reported an India ink sensitivity of 54.8%, which is close to the 62.5% observed in the present study. In the same investigation, LFA detected substantially more cases than conventional microscopy. These findings reinforce that a negative India ink result cannot reliably exclude cryptococcal meningitis and should be followed by a more sensitive antigen detection method when clinical suspicion persists [13]. In contrast, the CrAg LFA demonstrated 100% sensitivity, specificity, PPV, and NPV in both CSF and serum samples. Kabanda et al. similarly reported 100% sensitivity and specificity for CrAg LFA when applied to CSF specimens [14].

Gates-Hollingsworth and Kozel evaluated an early point-of-care CrAg lateral flow test using paired serum, plasma, and urine specimens from patients with active or recently treated cryptococcal meningitis. The assay showed strong agreement with quantitative antigen measurement and demonstrated the feasibility of rapid CrAg testing using different specimen types. This work provided important evidence supporting the development and implementation of lateral flow technology for decentralized cryptococcal diagnosis [15].

Overall, the findings of the present study support the incorporation of CrAg LFA into routine diagnostic algorithms for patients with suspected cryptococcal meningitis. Its excellent diagnostic accuracy, rapid turnaround time, ease of use, and applicability in resource-limited settings make it an ideal point-of-care test capable of facilitating early diagnosis and prompt initiation of antifungal therapy.

Limitations

The study included limited suspected meningitis cases, with few culture-confirmed cryptococcal meningitis cases. A larger sample size would provide more robust estimates of diagnostic accuracy. Since the study was conducted at a single tertiary care center, the findings may not be generalizable to other healthcare settings or different geographic regions.

## Conclusions

Based on the findings of this study, LFA should be strongly considered as the diagnostic method of choice for suspected cryptococcal meningitis, given its rapid turnaround, ease of use, and excellent diagnostic accuracy. While culture remains the definitive standard, its drawbacks in timely detection can be mitigated by the complementary or primary use of LFA, particularly in urgent or resource-limited settings. Adoption of these strategies, guided by comprehensive risk assessment, is likely to improve outcomes and reduce unnecessary delays in the treatment of cryptococcal meningitis.

## References

[1] Rajasingham R, Smith RM, Park BJ (2017). Global burden of disease of HIV-associated cryptococcal meningitis: an updated analysis. Lancet Infect Dis.

[2] (2022). Guidelines for diagnosing, preventing and managing cryptococcal disease among adults, adolescents and children living with HIV. https://www.who.int/publications/i/item/9789240052178.

[3] Perfect JR, Dismukes WE, Dromer F (2010). Clinical practice guidelines for the management of cryptococcal disease: 2010 update by the Infectious Diseases Society of America. Clin Infect Dis.

[4] Bicanic T, Harrison TS (2004). Cryptococcal meningitis. Br Med Bull.

[5] Perfect JR (2015). Cryptococcosis (Cryptococcus neoformans and Cryptococcus gattii). Mandell, Douglas, and Bennett's Principles and Practice of Infectious Diseases.

[6] Jarvis JN, Percival A, Bauman S (2011). Evaluation of a novel point-of-care cryptococcal antigen test on serum, plasma, and urine from patients with HIV-associated cryptococcal meningitis. Clin Infect Dis.

[7] Hansen J, Slechta ES, Gates-Hollingsworth MA (2013). Large-scale evaluation of the immuno-mycologics lateral flow and enzyme-linked immunoassays for detection of cryptococcal antigen in serum and cerebrospinal fluid. Clin Vaccine Immunol.

[8] Boulware DR, Rolfes MA, Rajasingham R (2014). Multisite validation of cryptococcal antigen lateral flow assay and quantification by laser thermal contrast. Emerg Infect Dis.

[9] Kozel TR, Bauman SK (2012). CrAg lateral flow assay for cryptococcosis. Expert Opin Med Diagn.

[10] Maziarz EK, Perfect JR (2016). Cryptococcosis. Infect Dis Clin North Am.

[11] Williamson PR, Jarvis JN, Panackal AA, Fisher MC, Molloy SF, Loyse A, Harrison TS (2017). Cryptococcal meningitis: epidemiology, immunology, diagnosis and therapy. Nat Rev Neurol.

[12] Kung V, Henao-Martínez AF, Tagawa A (2022). Diabetes mellitus as a risk factor for cryptococcosis - a multicenter research network study. Open Forum Infect Dis.

[13] Chisale MR, Salema D, Sinyiza F, Mkwaila J, Kamudumuli P, Lee HY (2020). A comparative evaluation of three methods for the rapid diagnosis of cryptococcal meningitis (CM) among HIV-infected patients in Northern Malawi. Malawi Med J.

[14] Kabanda T, Siedner MJ, Klausner JD, Muzoora C, Boulware DR (2014). Point-of-care diagnosis and prognostication of cryptococcal meningitis with the cryptococcal antigen lateral flow assay on cerebrospinal fluid. Clin Infect Dis.

[15] Gates-Hollingsworth MA, Kozel TR (2013). Serotype sensitivity of a lateral flow immunoassay for cryptococcal antigen. Clin Vaccine Immunol.

